# The impact of pandemic mental cognition on cultural values: an empirical study based on social media

**DOI:** 10.1186/s12889-023-16006-x

**Published:** 2023-06-05

**Authors:** Liuling Mo, Yun Liu, Ang Li, Tianli Liu, Tingshao Zhu

**Affiliations:** 1 Institute of Psychology, Chinese Academy of Sciences, Beijing, 100101 China; 2 Department of Psychology, University of Chinese Academy of Sciences, Beijing, 100049 China; 3 Dalian Vocational &Technical College, Dalian, 116035 China; 4 Department of Psychology, Beijing Forestry University, Beijing, 100083 China; 5 Institute of Population Research, Peking University, Beijing, 100871 China

**Keywords:** COVID-19, Pathogen stress hypothesis, Pandemic mental cognition, Sense of uncertainty, Cultural values

## Abstract

**Background:**

COVID-19 has triggered a global public health crisis, and had an impact on economies, societies, and politics around the world. Based on the pathogen prevalence hypothesis suggested that residents of areas with higher infection rates are more likely to be collectivists as compared with those of areas with lower infection rates. Many researchers had studied the direct link between infectious diseases and individualism/collectivism (infectious diseases→ cultural values), but no one has focused on the specific psychological factors between them: (infectious diseases→ cognition of the pandemic→ cultural values). To test and develop the pathogen prevalence hypothesis, we introduced pandemic mental cognition and conducted an empirical study on social media (Chinese Sina Weibo), hoping to explore the psychological reasons behind in cultural value changes in the context of a pandemic.

**Methods:**

We downloaded all posts from active Sina Weibo users in Dalian during the pandemic period (January 2020 to May 2022) and used dictionary-based approaches to calculate frequency of words from two domains (pandemic mental cognition and collectivism/individualism), respectively. Then we used the multiple log-linear regression analysis method to establish the relationship between pandemic mental cognition and collectivism/individualism.

**Results:**

Among three dimensions of pandemic mental cognition, only the sense of uncertainty had a significant positive relationship with collectivism, and also had a marginal significant positive relationship with individualism. There was a significant positive correlation between the first-order lag term AR(1) and individualism, which means the individualism tendency was mainly affected by its previous level.

**Conclusions:**

The study found that more collectivist regions are associated with a higher pathogen burden, and recognized the sense of uncertainty as its underlying cause. Results of this study validated and further developed the pathogen stress hypothesis in the context of the COVID-19 pandemic.

## Introduction

COVID-19 has triggered a global public health crisis which exerts economic, social, and political effects worldwide [1]. As COVID-19 spread globally, social distancing, self-isolation and national lockdowns have become crucial to control the pandemic [2–4]. Since the pandemic began, people’s maladjustment and emotion dysregulation increased, which may lead to changes in cultural values [5, 6]. The pathogen threats influence regional differences in cultural values since behavioral practices associated with cultural values may limit the spread of infectious diseases [7]. For example, the pathogen stress hypothesis (i.e., parasite stress model) suggested that more collectivist countries are associated with a higher pathogen burden [8–10]. An empirical study based on social media found an increased collectivism-related expression, and decreased individualism-related expression in China during the outbreak of the pandemic [11], indicating that people’s cultural values will be affected and change in the context of a pandemic, and the results support the pathogen hypothesis.

Individualism and collectivism have been considered as two vital and distinct cultural values for human society [12, 13]. Higher levels of individualism is defined as more focus on individual self, increased need for uniqueness (as opposed to conformity) and relatively weak family ties, while higher levels of collectivism is defined as more focus on intimate relationships, increased desire to fit into environment and hide individuality, and relatively strong family ties [14]. Early studies considered collectivism and individualism as two opposing cultural values [15]. However, recent studies identified the relationship of such two cultural values as orthogonal [18], and suggested the coexistence of individualism and collectivism in cultures and individuals [12, 16, 17]. Previous studies showed that collectivism represents a protective factor against negative emotions [6] and contributes much to people’s happiness [19], while individualism has a negative relationship with mental health [20, 21] but a positive relationship with scientific and technological innovation [22].

The COVID-19 outbreak has provided an opportunity to study the pathogen hypothesis, and many researchers have used the opportunity created by the pandemic to study the direct link between infectious diseases and individualism/collectivism (infectious diseases→ cultural values) [11, 23], but no one has focused on the specific psychological factors between them: (infectious diseases→ cognition of the pandemic → cultural values). The “Behavioral Immune System” [24–26] proposes psychological mechanisms that involved as a means to minimize infection risk by triggering specific emotional and cognitive responses to promote pathogen avoidance behaviors [10]. Accordingly, COVID-19 might trigger disease-relevant cognition, resulting in specific behaviors to avoid the physical and mental health risks. Just as individual cognitions change in response to infectious diseases, group-level value systems change in response to disease threats in local ecosystems [22]. On that basis, this paper proposes that mental cognitions of the pandemic may be responsible for adaptive changes in cultural values, thus protecting people from physical or psychological harm during the pandemic.

In this study, according to the Mental Cognition Scale [27], the pandemic mental cognition was characterized by three dimensions: behavioral protection tendency, positive attitude, and sense of uncertainty. Such three dimensions distinguished people’s mental cognition in the face of the pandemic from the level of behavioral tendency, attitudes and emotion. We intend to examine the effects of specific mental cognition dimensions on cultural values.

During COVID-19, social isolation led to a significant increase in people’s exposure to social media [28]. Due to home quarantine, people spent more time online to obtain information about the pandemic and record their living conditions. As a non-invasive analytical method, big data analytics has been proven to be effective in using social media behavioral data (e.g., posts, comments, and replies) to measure users’ emotions, cultural values and behavioral intentions [29–31]. In China, Sina Weibo is the most influential social media [32], with 530 million monthly active users and 230 million daily active users. Therefore, in this paper, Sina Weibo was selected as social media analysis platform. Moreover, since Dalian is a vital port city in China that has experienced multiple waves of COVID-19, compared with other cities, it is more suitable to explore the change of cultural value tendency during the pandemic in Dalian. Accordingly, data from users in Dalian were collected and analyzed.

**Fig. 1:**
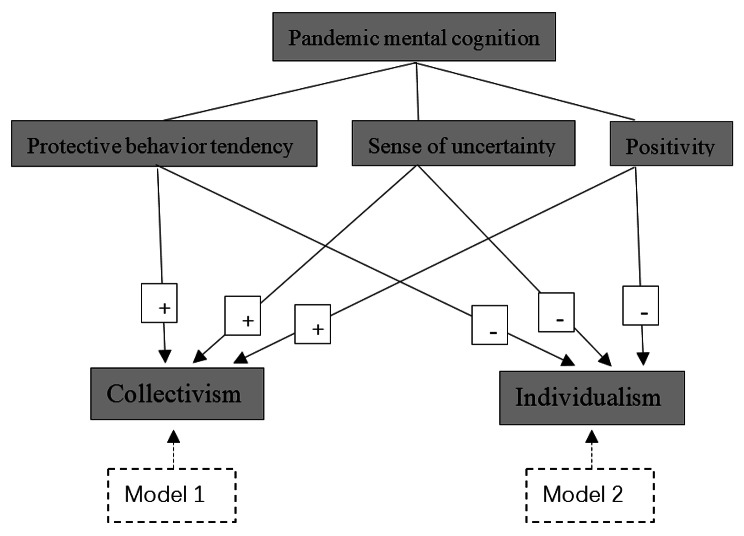
The models of research hypotheses

In the present study, we aimed to explore whether the pandemic mental cognition of people in Dalian might affect their cultural value tendency during COVID-19. We hypothesized that the pandemic mental cognition can positively predict collectivistic tendency and negative predict individualistic tendency (Fig. 1). We discussed the psychological factors behind the pathogen hypothesis in the study, hope to develop the pathogen hypothesis and provide an empirical basis for the causes of cultural values.

## Methods

Participants and data collection.

**Fig. 2:**
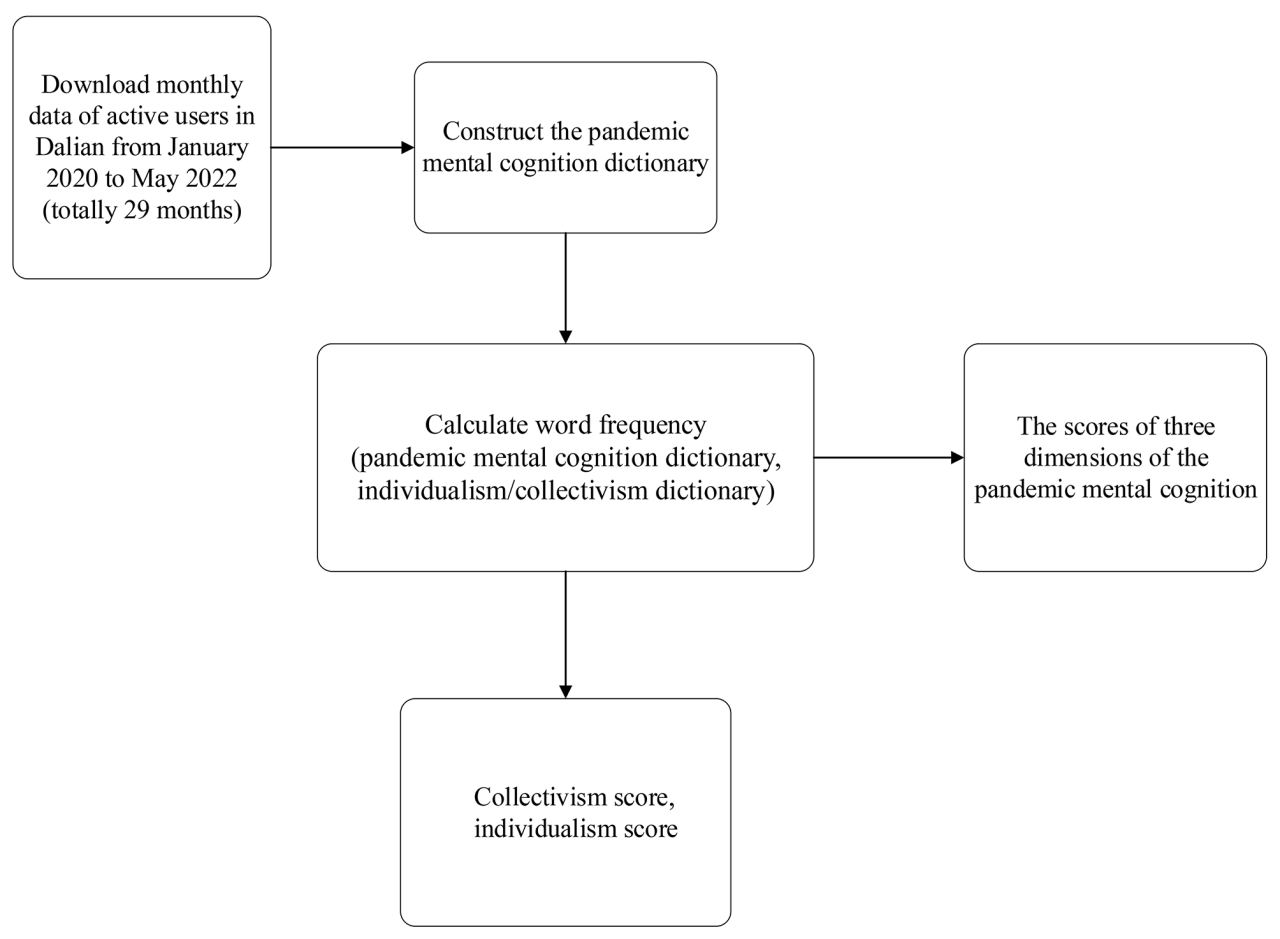
The procedure from data collection to calculation of psychological indicators

In this study, active Weibo users in Dalian were collected from Sina Weibo (https://weibo.com/newlogin?tabtype=weibo&gid=102803&openLoginLayer=0&url=https%3 A%2 F%2Fweibo.com%2 F.). We downloaded monthly data (Weibo posts) from active users in Dalian during the pandemic from January 2020 to May 2022 (totally 29 months). Then we used dictionary-based approaches to calculate frequency of words in Weibo posts from two domains (pandemic mental cognition and collectivism/individualism) respectively, month by month. Finally, we got the time series data of word frequency for all variables(Fig. 2).

Downloading Weibo data: The Weibo Application Programming Interface (API) of Sina Weibo is used to crawl the Weibo posts published by users in Dalian from January 2020 to May 2022, month by month.

Constructing the pandemic mental cognition dictionary: Following the Mental Cognition Scale [25], this study selected the keywords related to the three dimensions of mental cognition from the Sina Microblog during the COVID-19. After discussion by the panel of experts, we got the pandemic mental cognition dictionary showed in Table 1.

**Table 1:** The pandemic mental cognition dictionary

Protective behavior tendency	The intention of individuals to adopt self-protective behavior in order to reduce the potential health damage caused by the pandemic
8 keywords	Wear masks, disinfection, epidemic prevention, do not gather, no parties, no dinners, no going out, wash hands.
**Sense of uncertainty**	**A perception that arises when a person is unable to give proper classification or organization to an event or situation due to conflicts arising from lack or excess of information. In this paper, the sense of uncertainty refers to a subjective feeling and response caused by an individual’s inability to make a reasonable judgment on the epidemic.**
20 keywords	Worry, anxiety, confusion, have no idea what to do, Endless, hesitate, helpless, have no choice Unknown, devastated, overwhelmed, scared, in a state of anxiety, trembling, terrified, panic-stricken, mentally disturbed, fear, panic.
**Positivity**	**During the epidemic, people are not lying flat, but they are demanding in their thoughts and have relatively positive behaviors.**
8 keywords	Exercise regularly, keep exercising, keep exercising, live a regular life, take up interests, take up hobbies, study food, live an active life.

Calculating word frequency: We conducted Chinese word segmentation on the Weibo texts and removed all stop words. Then, we calculated word frequency using pandemic mental cognition dictionary (Table 1) and individualism/collectivism dictionary [30].

### Statistical analysis

SPSS was used to preliminary examine correlations between pandemic mental cognition and collectivism/individualism. Eviews, a statistical software suitable for the analysis of time series data, was used for multiple log-linear regression analysis.

Semi-log regression model was used to establish relationships between dimensions of pandemic mental cognition and collectivism/individualism, the equation is written as:

$$\text{l}\text{n}\text{y}={b}_{0}+{b}_{1}{x}_{1}+{b}_{2}{x}_{2}+{b}_{3}{x}_{3}+\in$$

where $\text{y}$ (collectivism/individualism) is the dependent variable, and ${x}_{1}$ (protective behavior tendency), ${x}_{2}$ (sense of uncertainty), ${x}_{3}$_(_positivity) are the independent variables used to predict $\text{y}$. The coefficients ${b}_{1}$, ${b}_{2}$,${b}_{3}$ describe the size of the effects of the independent variables on the dependent variable, and ${b}_{0}$ (also known as the intercept) is the predicted value of $\text{y}$ when all the independent variables are equal to 0, it can be seen as systematic error. And $\in$ is residuals, it can be seen as random error.

## Results

### Pearson correlation analysis

Table 2 showed that the sense of uncertainty was positively correlated with collectivism (r = 0.63, p < 0.01) and individualism (r = 0.54, p < 0.01). No other significant correlations were found.

**Table 2:** Pearson correlation coefficients between variables (n = 29)

	1	2	3	4	5
1 protective behavior tendency	1				
2 positivity	0.19	1			
3 sense of uncertainty	0.17	0.26	1		
4 collectivism	0.03	0.20	0.63^**^	1	
5 individualism	-0.01	0.21	0.54^**^	0.13	1

** p < 0.01

### Multiple log-linear regression analysis

- We found that the distributions of collectivism and individualism were not normal (Table 3), so the *ln* function was used to normalize values of them. At the same time, the logarithmic transformation of the dependent variable is also a feasible way to make the independent variable and the dependent variable become linear.
  After the normalization of collectivism/individualism, there were linear relationships between independent variables and dependent variables, and the residuals conformed to the normal distribution and homoscedasticity. However, there still existed autocorrelation (DW = 1.30, n = 29; DW = 0.87, n = 29) (see Table 4).
  Because the logarithmic transformation only for the dependent variable and not for the independent variable, we call the model a semi-log regression model. But due to autocorrelation, it was a biased model. Next, we need to eliminate the effect of autocorrelation on the model.
- Autocorrelation is an important concept of time series data, which means that the value of one moment in the time series has a certain correlation with the value of another moment in time. Since our data were time series data downloaded month by month, we choose Eviews for the analysis, and established semi-log regression models to examine the effect of three dimensions of pandemic mental cognition on collectivism/individualism. Besides, In order to separate the autocorrelation, the first-order lag term AR(1) and the second-order lag term AR(2) were also added as the independent variable in the regression model. The addition of AR terms is the use of the Cochrane-Orcutt iterative method to eliminate autocorrelation, when we establish a model with the least squares method.

**Table 3:** the normality test results

	Shapiro-Wilk test		
	t	df	p
Protective behavior tendency	0.66	29	0.00
positivity	0.89	29	0.01
sense of uncertainty	0.93	29	0.04
collectivism	0.92	29	0.03
individualism	0.92	29	0.03

**Table 4:** Multiple log-linear regression results after the normalization of collectivism/individualism

	Model 1			Model 2			
Variable	bi	p		bi	p	tolerance value	VIF
Protective behavior tendency	-66.83	0.65		-141.21	0.51	0.95	1.06
Positivity	1069.05	0.92		9018.99	0.573	0.91	1.10
Sense of uncertainty	1323.02	0.00		1666.87	0.00	0.92	1.09
F	5.00	0.01		4.10	0.02		
R^2^	0.38			0.33			
AdjustedR^2^	0.30			0.25			
DW	1.30			0.87			

The dependent variable of model 1 is collectivism

The dependent variable of model 2 is individualism

The results of multiple log-linear regression were shown in Table 5. Overall, the semi-log regression model of collectivism has a good overall fit, which could explain 44% of variation in the dependent variable (R^2^ = 0.44, adjusted R^2^ = 0.31, p < 0.05). Moreover, the autocorrelation problem no longer existed in the model (DW = 2.04, n = 29). The semi-log regression model of individualism has a good performance, which could explain 62% of variation in the dependent variable (R^2^ = 0.62, adjusted R^2^ = 0.52, p < 0.001). Besides, the autocorrelation problem no longer existed in the model (DW = 2.13, n = 29).

Specifically, the linear relationship between behavior protection tendency and collectivism was not significant (β=-1.12, p > 0.05); and the linear relationship between positive attitude and collectivism was not significant as well (β = 2051.68, p > 0.05). Only the sense of uncertainty had a significant positive relationship with collectivism (β = 1345.89, p < 0.05). The results indicated that, among three dimensions of pandemic mental cognition, only the sense of uncertainty about the pandemic increases the collectivism-related expression of users in Dalian.

the relationship between behavior protection tendency and individualism was not significant (β=-272.43, p > 0.05), and the relationship between positive attitude and individualism was not significant as well (β=-8951.92, p > 0.05). There was a marginal significant positive correlation between the sense of uncertainty and individualism (β = 1342.84, p = 0.05). The first-order lag term AR(1) was significant (β = 0.93, p < 0.001).

The results indicate that there existed first order autocorrelation, which is a type of serial correlation. It occurs when there is a correlation between successive errors. In it, errors of the one-time period correlate with the errors of the consequent time period. It means that individualism tendency was mainly affected by its previous level, the last month’s level of individualism can positively predict people’s current individualism tendency. Besides, among three dimensions of pandemic mental cognition, only the sense of uncertainty about the pandemic may increase the individualism tendency.

**Table 5:** Multiple log-linear regression analysis results

	Model 1			Model 2			
Variable	bi	p		bi	p	tolerance value	VIF
Protective behavior tendency	-1.12	0.99		-272.43	0.22	0.95	1.06
Positivity	2051.68	0.85		-8951.92	0.45	0.91	1.10
Sense of uncertainty	1345.89	0.02		1342.84	0.05	0.92	1.09
AR(1)	0.34	0.31		0.93	0.00		
AR(2)				-0.30	0.34		
F	3.56	0.02		6.10	0.00		
R^2^	0.44			0.62			
AdjustedR^2^	0.31			0.52			
DW	2.04			2.13			

The dependent variable of model 1 is collectivism

The dependent variable of model 2 is individualism

## Discussion

In the present study, we found that the behavioral protection tendency and positivity of pandemic mental cognition didn’t affect the cultural values, while the sense of uncertainty about pandemic positively predicted people’s collectivism expression on social media, and may also have a positive predictive effect on individualism expression. However, the individualism tendency was primarily affected by its previous level, rather than by the mentality cognition of the pandemic.

Specifically, in the present research, it was found that the sense of uncertainty about the pandemic had a positive effect on collectivism, which may be because the uncertainty about the pandemic would make people feel fear, and fear can trigger people’s collectivism tendency [33]. At the same time, regardless of concerns about the risk of infection, people with a sense of belonging and connection with others can best cope with the threat of isolation [34–36]. Therefore, it’s reasonable that people would generate more collectivistic expression when they feel uncertainty about the pandemic. Another hypothesis is that disasters reduce individual agency and individual autonomy, which strengthen their need to rely on others [37]. So people may show greater collectivism tendency and generate more collectivistic expression when faced with risks.

The results suggest that the sense of uncertainty may trigger more collectivistic tendency for more social support and security. Meanwhile, in order to reduce people’s sense of uncertainty, it should emphasize the importance of regularly releasing substantive official updates and monitoring social media during crisis events to reduce exposure to misleading information and confusions [38]. In addition, the pathogen hypothesis considered that the development of cultural values takes a long period of time, and our results further explored the possible psychological factors behind the changes in cultural values, which enriched the research on the pathogen hypothesis.

Contrary to our hypothesis, pandemic mental cognition didn’t negatively predict individualism. Specifically, we found that the tendency of individualism was not affected by the pandemic mental cognition, but mainly affected by the previous level of individualism. The sense of uncertainty had a marginal positive effect on the prediction of individualism tendency. The possible reason may be that China performed social distancing as a public health tool to control the pandemic, and such acts could make people feel isolated. The sense of isolation may be partly responsible for sense of uncertainty about the outbreak. Notably, individualism helps to reduce the fear of isolation [39]. Thus, the sense of isolation during the pandemic may trigger individualistic tendency and leading to increased individualistic expressions.

Previous study found that individualism was positively (rather than negatively) correlated with the frequency of disasters [40]. This may be because the anxiety and stress caused by the disaster made people pay less attention to social background information [41], so they pay more attention to their own feelings. In this study, in order to gain more sense of control and security, the sense of uncertainty for pandemic may also make people turn their focus from the outside to their internal feelings. This point provides us a new perspective, that is, individualism may also serve as protective factor against negative emotions.

The global pandemic had a strong negative impact on everyone, with increased feelings of uncertainty, insecurity, current and future instability, as well as decreased feelings of autonomy and self-direction [42, 43]. The results of our study suggest a new direction for mental health intervention during the pandemic period: we should encourage people to maintain connections with others, and develop the sense of control over their lives to reduce the sense of uncertainty about unknown risks.

We collected and analyzed large-scale data from Sina Weibo, to test pathogen stress hypothesis, which can overcome difficulties in tracing the changes of cultural values. In addition, this study found the psychological factors behind the pathogen hypothesis, and provided an empirical basis for the causes of cultural values, which developed the pathogen hypothesis.

Limitations also existed in this study. Specifically, this study mainly focused on Weibo users in Dalian, and Weibo has a relatively high proportion of young users, so the sample of this study may not be a good representation of all people in Dalian. In addition, the sample of our study came from a port city with collectivism cultural context, caution is necessary when generalizing the conclusions to other case. In future research, we can focus on more psychological factors to help people better respond to public health crises.

## Conclusion

Among the three dimensions of pandemic mental cognition, only the sense of uncertainty affects collectivism tendency. Besides, the individualism tendency was mainly affected by its previous level. In our study, the pathogen prevalence hypothesis in the context of COVID-19 was further validated and developed. The results not only suggest that people in areas with higher infection rates are more likely to be collectivists, but also recognize the sense of uncertainty as its underlying cause.

## Data Availability

The datasets used and/or analyzed during the current study are available from the corresponding author on reasonable request.

## References

[1] Mehta P, McAuley DF, Brown M, Sanchez E, Tattersall RS, Manson JJ (2020). COVID-19: consider cytokine storm syndromes and immunosuppression. Lancet (London England).

[2] Parmet WE, Sinha MS (2020). Covid-19 - the Law and limits of Quarantine. N Engl J Med.

[3] Prem K, Liu Y, Russell TW, Kucharski AJ, Eggo RM, Davies N, Jit M, Klepac P (2020). The effect of control strategies to reduce social mixing on outcomes of the COVID-19 epidemic in Wuhan, China: a modelling study. The Lancet Public health.

[4] Viner RM, Russell SJ, Croker H, Packer J, Ward J, Stansfield C, Mytton O, Bonell C, Booy R (2020). School closure and management practices during coronavirus outbreaks including COVID-19: a rapid systematic review. The Lancet Child & adolescent health.

[5] Fincher CL, Thornhill R, Murray DR, Schaller M (2008). Pathogen prevalence predicts human cross-cultural variability in individualism/collectivism. Proc Biol Sci.

[6] Kim HS, Sherman DK, Updegraff JA (2016). Fear of Ebola: the influence of Collectivism on xenophobic threat responses. Psychol Sci.

[7] Schaller M, Murray DR (2011). Infectious disease and the creation of culture. *Advances in culture and psychology, Vol. 1*; advances in culture and psychology.

[8] Fincher CL, Thornhill RA, Parasite-Driven, Wedge. Infectious Diseases May Explain Language and Other Biodiversity %J Oikos. 2008, *117*.

[9] Thornhill R, Fincher CL, Murray DR, Schaller M (2010). Zoonotic and non-zoonotic Diseases in Relation to Human personality and societal values: support for the parasite-stress model. Evolutionary Psychol.

[10] Murray DR, Trudeau R, Schaller M (2011). On the Origins of Cultural differences in conformity: four tests of the Pathogen Prevalence Hypothesis. Pers Soc Psychol Bull.

[11] Han N, Ren X, Wu P, Liu X, Zhu T (2021). Increase of Collectivistic expression in China during the COVID-19 outbreak: an empirical study on Online Social Networks. Front Psychol.

[12] Triandis HC. (1995). Individualism And Collectivism (1st ed.). Routledge. 10.4324/9780429499845.

[13] Hui CH, Triandis HC, Individualism-Collectivism (1986). A study of Cross-Cultural Researchers. J Cross-Cult Psychol.

[14] Grossmann I, Na J (2014). Research in culture and psychology: past lessons and future challenges. Wiley Interdiscip Rev Cogn Sci.

[15] Hofstede G (1980). Culture’s Consequences.

[16] Kim SS (2009). Individualism and collectivism: implications for women. Pastoral Psychol.

[17] Vargas JH, Kemmelmeier M (2013). Ethnicity and contemporary american culture: a meta-analytic investigation of horizontal–vertical individualism–collectivism. J Cross-Cult Psychol.

[18] Vargas JH, Kemmelmeier M (2012). Ethnicity and contemporary American Culture: a Meta-Analytic Investigation of Horizontal–Vertical individualism–collectivism. J Cross-Cult Psychol.

[19] Ahuja KK, Banerjee D, Chaudhary K, Gidwani C (2021). Fear, xenophobia and collectivism as predictors of well-being during coronavirus disease 2019: an empirical study from India. Int J Soc Psychiatry.

[20] Zandi GK, Mostafa M, Taleb H. Individualism and Mental Health: A Study of People of Kohgiluyeh and Boyer-Ahmad Province, Iran %J World Family Medicine Journal/Middle East Journal of Family Medicine. 2018, *16*.

[21] Ashley H, Maria BA, Pascal M. The social contract revisited: a re-examination of the influence individualistic and collectivistic value systems have on the psychological wellbeing of young people %J Journal of Youth Studies. 2020, *23*.

[22] Murray DR (2014). Direct and indirect implications of Pathogen Prevalence for Scientific and Technological Innovation. J Cross-Cult Psychol.

[23] Na J, Kim N, Suk HW, Choi E, Choi JA, Kim JH, Kim S, Choi I. Individualism-collectivism during the COVID-19 pandemic: a field study testing the pathogen stress hypothesis of individualism-collectivism in Korea. Pers Indiv Differ. 2021;183. 10.1016/j.paid.2021.111127. 10.1016/j.paid.2021.111127 PMC9757850 36569789

[24] Schaller M, Duncan LA. The behavioral immune system: its evolution and social psychological implications. Evol social mind: Evolutionary Psychol social cognition 2007, 293–307.

[25] Schaller M (2011). The behavioural immune system and the psychology of human sociality. Philos Trans R Soc Lond B Biol Sci.

[26] Schaller M, Parasites. Behavioral defenses, and the Social Psychological Mechanisms through which cultures are evoked. Psychol Inq. 2006;17. 10.1207/s15327965pli1702_2.

[27] Yun Liu TZ, Li A. Development and Test of Mental Cognition Scale for College Students in COVID-19. *CSTR: i>32003.36.ChinaXiv.202207.00008.V2* 2002.

[28] Gao J, Zheng P, Jia Y, Chen H, Mao Y, Chen S, Wang Y, Fu H, Dai J (2020). Mental health problems and social media exposure during COVID-19 outbreak. PLoS ONE.

[29] Dong Y, Chen H, Tang X, Qian W, Zhou A. Prediction of social mood on Chinese societal risk perception. In Proceedings of the 2015 International Conference on Behavioral, Economic and Socio-cultural Computing (BESC), 30 Oct.-1 Nov. 2015, 2015; pp. 102–108.

[30] Xiaopeng REN, Yuanyuan X, Yang Z, Tingshao ZHU (2017). Individualism/collectivism map of China based on Weibo. J Inner Mongolia Normal Univ (Philosophy Social Science).

[31] Hernández-García I, Giménez-Júlvez T. Characteristics of YouTube videos in spanish on how to prevent COVID-19. 2020, *17*, 4671. 10.3390/ijerph17134671 PMC7370194 32610523

[32] Weibo Development Report Available online. : https://data.weibo.com/report/reportDetail?id=456&sudaref=cn.bing.com (accessed on 12 March 2021).

[33] Sverko D (2009). Emotions in the Context of Individualism and Collectivism Dimensions. DRUSTVENA ISTRAZIVANJA.

[34] Burson A, Crocker J, Mischkowski D (2012). Two types of Value-Affirmation: implications for self-control following Social Exclusion. Social Psychol Personality Sci.

[35] Shnabel N, Purdie-Vaughns V, Cook JE, Garcia J, Cohen GL (2013). Demystifying values-affirmation interventions: writing about social belonging is a key to buffering against identity threat. Personal Soc Psychol Bull.

[36] Triandis H. C.J.J.o.p. Individualism-collectivism and personality. 2001, *69 6*, 907–24. 10.1111/1467-6494.696169 11767823

[37] Triandis HC. Ecological determinants of cultural variation. 2009.

[38] Purgato M, Gastaldon C, Papola D, van Ommeren M, Barbui C, Tol WA. Psychological therapies for the treatment of mental disorders in low- and middle-income countries affected by humanitarian crises. Cochrane Database Syst Rev. 2018;7(Cd011849). 10.1002/14651858.CD011849.pub2. 10.1002/14651858.CD011849.pub2 PMC6513488 29975811

[39] Hong Seong CA, Cross-Cultural (2020). Study of the spiral of silence theory with individualism-collectivism and uncertainty-avoidance. The Korea Contents Association.

[40] Grossmann I, Varnum MEW, Social Structure (2015). Infectious Diseases, Disasters, Secularism, and Cultural Change in America. Psychol Sci.

[41] Wachtel PL (1968). Anxiety, attention, and coping with threat. J Abnorm Psychol.

[42] Ornell F, Schuch JB, Sordi AO, Kessler FHP. “Pandemic fear” and COVID-19: mental health burden and strategies. *Revista brasileira de psiquiatria (Sao Paulo, Brazil*: 1999) 2020, *42*, 232–235, doi:10.1590/1516-4446-2020-0008. 10.1590/1516-4446-2020-0008 PMC7236170 32267343

[43] Torales J, O’Higgins M, Castaldelli-Maia JM, Ventriglio A (2020). The outbreak of COVID-19 coronavirus and its impact on global mental health. Int J Soc Psychiatry.

